# Cell Model for Testing Pharmaceuticals Targeting Human PD-L1

**DOI:** 10.17691/stm2024.16.5.01

**Published:** 2024-10-30

**Authors:** O.A. Shashkova, L.A. Terekhina, I.S. Malakhov, A.A. Pinevich, N.L Vartanyan, K.O. Avrov, I.Yu. Krutetskaya, I.V Gryazeva, M.A. Berlina, A.Yu. Stolbovaya, I.V. Smirnov, S.V. Fedorenko, A.A. Krylova, M.A. Nadporojskii, S.V Shatik, A.A. Stanzhevskii, M.P. Samoilovich

**Affiliations:** PhD, Senior Researcher, Hybridoma Technology Laboratory; A.M. Granov Russian Research Center for Radiology and Surgical Technologies, Ministry of Health of the Russian Federation, 70 Leningradskaya St., Saint Petersburg, Pesochniy pos., 197758, Russia; Researcher, Hybridoma Technology Laboratory; A.M. Granov Russian Research Center for Radiology and Surgical Technologies, Ministry of Health of the Russian Federation, 70 Leningradskaya St., Saint Petersburg, Pesochniy pos., 197758, Russia; Senior Researcher, Hybridoma Technology Laboratory; A.M. Granov Russian Research Center for Radiology and Surgical Technologies, Ministry of Health of the Russian Federation, 70 Leningradskaya St., Saint Petersburg, Pesochniy pos., 197758, Russia; Postgraduate Student, Institute of Virology and Cell Biology; University of Lübeck, 160 Ratzeburger Allee, Lübeck, 23562, Germany; PhD, Senior Researcher, Hybridoma Technology Laboratory; A.M. Granov Russian Research Center for Radiology and Surgical Technologies, Ministry of Health of the Russian Federation, 70 Leningradskaya St., Saint Petersburg, Pesochniy pos., 197758, Russia; Senior Lecturer, Cytology and Histology Department, Biological Faculty; Saint Petersburg State University, 7/9 Universitetskaya Naberezhnaya, Saint Petersburg, 199034, Russia; PhD, Senior Researcher, Hybridoma Technology Laboratory; A.M. Granov Russian Research Center for Radiology and Surgical Technologies, Ministry of Health of the Russian Federation, 70 Leningradskaya St., Saint Petersburg, Pesochniy pos., 197758, Russia; PhD, Senior Researcher, Hybridoma Technology Laboratory; A.M. Granov Russian Research Center for Radiology and Surgical Technologies, Ministry of Health of the Russian Federation, 70 Leningradskaya St., Saint Petersburg, Pesochniy pos., 197758, Russia; PhD, Senior Researcher, Hybridoma Technology Laboratory; A.M. Granov Russian Research Center for Radiology and Surgical Technologies, Ministry of Health of the Russian Federation, 70 Leningradskaya St., Saint Petersburg, Pesochniy pos., 197758, Russia; PhD, Senior Researcher, Hybridoma Technology Laboratory; A.M. Granov Russian Research Center for Radiology and Surgical Technologies, Ministry of Health of the Russian Federation, 70 Leningradskaya St., Saint Petersburg, Pesochniy pos., 197758, Russia; Laboratory Researcher, Hybridoma Technology Laboratory; A.M. Granov Russian Research Center for Radiology and Surgical Technologies, Ministry of Health of the Russian Federation, 70 Leningradskaya St., Saint Petersburg, Pesochniy pos., 197758, Russia; Researcher, Hybridoma Technology Laboratory; A.M. Granov Russian Research Center for Radiology and Surgical Technologies, Ministry of Health of the Russian Federation, 70 Leningradskaya St., Saint Petersburg, Pesochniy pos., 197758, Russia; PhD, Leading Researcher, Hybridoma Technology Laboratory; A.M. Granov Russian Research Center for Radiology and Surgical Technologies, Ministry of Health of the Russian Federation, 70 Leningradskaya St., Saint Petersburg, Pesochniy pos., 197758, Russia; Engineer of the 1^st^ Category, Physical and Technical Support Group of Radiation Therapy; A.M. Granov Russian Research Center for Radiology and Surgical Technologies, Ministry of Health of the Russian Federation, 70 Leningradskaya St., Saint Petersburg, Pesochniy pos., 197758, Russia; Laboratory Researcher, Hybridoma Technology Laboratory; A.M. Granov Russian Research Center for Radiology and Surgical Technologies, Ministry of Health of the Russian Federation, 70 Leningradskaya St., Saint Petersburg, Pesochniy pos., 197758, Russia; Researcher, Department of Cyclotron-Produced Radiopharmaceuticals; A.M. Granov Russian Research Center for Radiology and Surgical Technologies, Ministry of Health of the Russian Federation, 70 Leningradskaya St., Saint Petersburg, Pesochniy pos., 197758, Russia; PhD, Head of the Department of Cyclotron-Produced Radiopharmaceuticals; A.M. Granov Russian Research Center for Radiology and Surgical Technologies, Ministry of Health of the Russian Federation, 70 Leningradskaya St., Saint Petersburg, Pesochniy pos., 197758, Russia; DSc, Deputy Director of Research; A.M. Granov Russian Research Center for Radiology and Surgical Technologies, Ministry of Health of the Russian Federation, 70 Leningradskaya St., Saint Petersburg, Pesochniy pos., 197758, Russia; DSc, Chief Researcher, Head of the Hybridoma Technology Laboratory; A.M. Granov Russian Research Center for Radiology and Surgical Technologies, Ministry of Health of the Russian Federation, 70 Leningradskaya St., Saint Petersburg, Pesochniy pos., 197758, Russia; Chief Researcher, Cytology and Histology Department, Biological Faculty; Saint Petersburg State University, 7/9 Universitetskaya Naberezhnaya, Saint Petersburg, 199034, Russia

**Keywords:** PD-L1, radioconjugate, VHH, cell model, CT26, targeted agent, tumor model

## Abstract

**The aim of this study** was to create and evaluate a cell model designed for *in vitro* and *in vivo* testing of anti-human PD-L1 therapeutic and diagnostic agents’ specificity.

**Materials and Methods:**

Genetically modified cells expressing human PD-L1 (strain CT26-PD-L1) were obtained by retroviral transduction of murine CT26 carcinoma cells. *PD-L1* gene activity was assessed by real-time PCR, and PD-L1 expression on cells was identified by flow cytometry. Cells were tested using recombinant single-domain human anti-PD-L1 antibodies (nanoantibodies) conjugated with radioisotopes ^68^Ga or ^177^Lu. Immunoreactive fraction and cell internalization of the radioconjugates were evaluated *in vitro.* For *in vivo* experiments CT26-PD-L1 cells were transplanted into mice, radioimmunoconjugates were injected 9-14 days later, in 1-48 h the tumors were retrieved and subjected to direct radiometry. Intact CT26 cells not expressing the antigen served as a control.

**Results:**

CT26-PD-L1 strain of murine tumor cells expressing human membrane PD-L1 was created. When transplanted into intact BALB/c mice or sublethally irradiated F1(DBA×BALB/c) mice, these cells formed tumors. Thus, a significant advantage of the model was the possibility of *in vivo* testing of human PD-L1-affinity agents using animals under conventional vivarium conditions. When radioimmunoconjugates were administered to tumor bearing mice, radionuclides accumulated in tumors generated from the transplanted CT26-PD-L1 cells, but not CT26 cells. CT26-PD-L1 cells internalized anti-PD-L1 nanobodies *in vitro.* Due to a high density of target molecules, CT26-PD-L1 cells allowed both to confirm pharmaceuticals’ specificity and to quantify the target-binding fraction of conjugates in a single test.

**Conclusion:**

The created cells are the first genetically engineered cells designed to evaluate affinity of anti-human PD-L1 therapeutic and diagnostic agents in Russia. Test results confirmed the model suitability for *in vitro* and *in vivo* testing of the specificity of pharmaceuticals targeting human PD-L1.

## Introduction

From all molecules involved in the immune response regulation, the PD-1/PD-L1 receptor-ligand pair is of great significance. Binding of PD-1 receptor on T-lymphocytes with PD-L1 ligand of antigen-presenting cells results in inhibition of proliferation, cytokine production, and cytotoxic function of T-lymphocytes, which normally prevents autoimmune reactions and chronic inflammation, as well as limits a specific immune response during pregnancy [1–5]. The same mechanism is used by tumor cells to avoid immune response. PD-L1 is expressed by the cells of lung carcinoma, brain tumors, as well as tumors of thyroid gland, thymus, breast, gastrointestinal tract, liver, pancreas, kidneys, adrenal cortex, bladder, and ovaries, squamous cell carcinoma of the head and neck, and melanoma [4–8]. High PD-L1 expression is demonstrated by solid tumor cells resistant to radiation and chemical therapy [8–11]. Currently, PD-L1 is considered the main biomarker for targeted therapy of various nosological forms of cancer *ad hoc* to traditional treatment. However, there is still no standard preclinical model for *in vivo* and *in vitro* testing of specificity and efficacy of the developed drugs having affinity to this biomarker.

There are two strategies of anti-PD-L1 therapy (PD-L1-targeted therapy). The first is based on the use of antibodies, their fragments, and small molecules that specifically interact with PD-L1 and prevent its binding to PD-1, which facilitates reactivation of antitumor immune response. The second strategy is the use of affinity molecules for targeted delivery of radionuclides, toxins, cytostatic drugs, etc. to the tumor for destruction of PD-L1-expressing tumor cells [12]. The choice of the strategy determines the biological model for assessment of the pharmacological effect of a drug targeted at PD-L1. Testing of immunotherapeutic potential of drugs requires immunocompetent animals preferably with a humanized immune system, but the use of such animals is significantly limited due to complexity and high cost of obtaining such models. Assessment of the antitumor effect of targeted molecule conjugates with pharmacologically active ingredients can be conducted on animals with various immunological statuses. Here, simpler models are sufficient to confirm drug specificity and activity *in vivo*.

In recent years, non-invasive diagnostics of neoplastic processes using affinity molecules conjugated to radionuclides has become more significant. For this purpose, radiopharmaceuticals are developed to target different biomarkers and carry radioisotopes with various characteristics. During conjugation with radionuclides some affine molecules lose their ability to specifically interact with the target, as a result the efficacy of the radiopharmaceuticals decreases. Thus, the key stage in testing is the quantitative assessment of the target-binding fraction of the radioconjugate [13, 14]. For this purpose, several *in vitro* methods are used. For instance, a fast and reproducible method is based on the use of magnetic particles coated with biomarker molecules [14, 15]. However, due to different spatial configuration of such molecules on the membrane of tumor cells and on the surface of magnetic particles, additional confirmation of the targeted agent specificity on cells is required. Moreover, magnetic particles cannot be used for testing pharmaceuticals *in vivo*. Cultured lines of human tumor cells are used as cell models applicable for testing conjugates of affinity molecules *in vitro* and *in vivo*. However, the number of biomarker molecules on their membranes is usually low, which prevents quantitative assessment of the target-binding fraction of pharmaceuticals *in vitro* [14]. The need to use immunodeficient mice (BALB/c nude, SCID, NOD-SCID, etc.) limits the use of human cells *in vivo*. At the same time there is a direct correlation between the animals’ immunodeficiency and efficacy of tumor transplantation and growth [16]. The deeper the immunodeficiency, the higher the cost of such animals, their maintenance, and experiments.

Recently, alternative models have included murine tumor cells that carry human biomarkers created by genetic engineering. Depending on the properties of the biomarker molecules and the murine tumor cells, mice with various immunological statuses including fully immunocompetent animals can serve as recipients of such humanized cells. This approach has already been approved abroad. It is confirmed by scientific publications, as well as commercial offers of cultured murine cells carrying human biomarkers [17–32]. As far as we know, such genetically engineered cell models designed for *in vitro* and *in vivo* testing of pharmaceuticals with affinity to human PD-L1 have not yet been created in Russia.

**The aim of this study** was to create and evaluate a cell model designed for *in vitro* and *in vivo* testing of anti-human PD-L1 therapeutic and diagnostic agents’ specificity.

## Materials and Methods

### Cell lines and culturing conditions

Human tumor cell lines of various histogenesis (NC-37, IM-9, RPMI 1788, T2, T24, U-2 OS, R1, EA.hy926, A172, Capan-2, U937, HEPG2, RD, MIA PaCa-2, U266, Kg-1, THP-1, A549, Jurkat, Namalwa, MeWo, CaCo2, Mg-63, SK-N-MC, MCF7, T98G, HL-60, Y79) and the Platinum-E packaging cell line for retroviral transduction (Cell Biolabs Inc., USA) were used in the study. Murine CT26 cells (BALB/c murine colon carcinoma) were used to create humanized cells. All cell cultures were stored in the laboratory’s cryobank and tested to confirm non-contamination with mycoplasmas, bacteria, and fungi. Cells were cultured in plates and plastic flasks (Jet Biofl, China; Orange Scientific, Belgium; Nunc, Denmark) in thermostats at 37°C, 6% CO_2_, and 100% humidity. Monolayer cultures were grown in DMEM/ F12, DMEM/Glucose 4.5 g/l/HEPES medium with the addition of L-glutamine (PanEco, Russia) or α-MEM (BioloT, Russia), suspension cultures were grown in RPMI-1640 medium (PanEco, Russia). The complete medium contained 5–10% fetal bovine serum (BioWest, France). For stable production of retroviral particles, Platinum-E cells were grown with selective antibiotics: 1 μg/ml of puromycin and 10 μg/ml of blasticidin. In case of monolayer cultures, cells were removed from the surface using a 0.25% trypsin solution with EDTA (BioloT, Russia).

### Pharmaceuticals targeting human PD-L1

Human PD-L1 affine molecules were recombinant single-domain heavy-chain antibodies (nanoantibodies, VHH) (Innova Plus, Russia) with a molecular weight of 10–15 kDa, synthesized in *Escherichia coli*. We used them as the basis for preparation of radioconjugates using ^68^Ga ([^68^Ga]Ga-VHH-PD-L1) or ^177^Lu ([^177^Lu]Lu-VHH-PD-L1). The substances radiochemical purity was >95%.

### PCR

Total RNA was isolated from cells using TRIzol Reagent (Life Technologies, USA) according to the manufacturer’s recommendations. Based on the obtained RNA, cDNA was synthesized by reverse transcription using random primers and M-MuLV RNase murine leukemia virus reverse transcriptase (SibEnzyme, Russia) according to the enzyme manufacturer’s recommendations. The synthesized cDNA was used to assess human *PD-L1* gene activity in cells by real-time polymerase chain reaction using forward (TGGCATTTGCTGAACGCATTT) and reverse (TGCAGCCAGGTCTAATTGTTTT) primers. The gene activity level was presented as the difference (Δ*Ct*) between the threshold cycle of *PD-L1* gene and *GAPDH* comparison gene, where *Ct* is the threshold cycle corresponding to the number of amplification cycles required to achieve the fuorescence threshold value.

### Generation of a murine tumor cell line expressing membrane human PD-L1

The donor of *PD-L1*-encoding nucleotide sequence was selected from human cell cultures stored in laboratory’s cryobank. *PD-L1* cDNA was amplified using forward (ATGTCT**GCGGCCGC**Catgggtgtcaaggtattatttgccctgata tgcattgctgtggcagaggcaTTTACTGTCACGGTTC) and reverse (ATTACT**GAATTC**GATCAGAAGTTCCAATG CTGG) primers (nuclease restriction sites are bolded; lowercase letters indicate the introduced signal sequence of *Gaussia princeps* luciferase for optimal expression of the target protein; *PD-L1* complementary sequences are underlined). The resulting sequence was cloned to the pQCXIP retroviral vector (Clontech, USA) containing the puromycin (selective antibiotic) resistance gene. The vector was used to transfect Platinum-E packing cells by calcium phosphate method. The culture fluid containing retroviral particles was used to transduce CT26 cells. Selection of cells stably expressing membrane PD-L1 was performed by adding puromycin to the growth medium.

### Flow cytometry

PD-L1 expression on cell membranes was identified by flow cytometry using phycoerythrin-labeled antibodies to human PD-L1 (BioLegend, USA). Cells were analyzed on BD FACS AriaIII flow cytometer using BD FACS Diva software version 7.0 (BD Biosciences, USA).

### Cell culture growth rate

To determine the cell culture growth rate, the cell doubling time (*Td*) was calculated by the following formula: *Td*=*dt*·ln2/ln(*N*/*N*_0_), where *dt* is the cell culturing time from seeding to culture removal (h), *N* is the number of cells at culture removal, *N*_0_ is the number of cells at culture seeding. The number of cells was estimated using Z1 Coulter Counter conductometric counter (Beckman Coulter, USA).

### Internalization of radioconjugates by cells

The cells were grown in culture flasks until 70% confluency was achieved. Then growth medium was completely replaced and the nanoantibody-based radioconjugate was added to the cell monolayer (0.1 μg nanoantibodies per 2 million cells, 50 kBq). The cells were incubated for 30 or 100 min at 37°C. After the specified time, the culture fluid was collected and the cells were washed twice with phosphate-buffered saline to remove unbound radioconjugate. Radioconjugate fraction bound to the membrane receptor was obtained by incubating the cells with 50 mM glycine solution in phosphate-buffered saline containing 0.1 M NaCl (pH 2.8). The internalized fraction was collected after cell lysis using 1N NaOH solution followed by neutralization with 1N HCl. Radioactivity of the obtained fractions was identified using Triathler radiometer (Hidex Oy, Finland). All radioactivity values were recalculated for the time of the first sample measurement (zero point) using the following formula: *A_0_=A_t_·e^λt^*, where *A_0_* is the radioactivity of the sample at zero point, *A_t_* is the radioactivity of the sample measured *t* minutes after the start of measurement (measurement of the first sample), λ is the radioactive decay constant. The fraction volume was expressed as a percentage of the total radioactivity added to the cells.

### Target-binding (immunoreactive) fraction of radio-conjugates

The target-binding fraction of radio conjugates was identified by incubating radiolabeled nanoantibodies (at a constant concentration of 0.25 μg/ml) with antigen-expressing cells in various concentrations (0.3-20 million/ml). The cells were then washed from unbound radioconjugate, and radioactivity was determined in all samples as specified above. The immunoreactive fraction was calculated as the ratio of cell-bound radioactivity (*B*) to the initially added radioactivity (*T*) when the antigen’s excess was reached; the resulting value was expressed as a percentage.

### Model of murine tumor expressing human PD-L1

*In vivo* experiments were conducted on BALB/c and F1(DBA×BALB/c) mice of both genders maintained under standard conditions in accordance with Directive 2010/63/EU of the European Parliament and the EU Council “On Protection of Animals Used for Scientific Purposes” dated September 22, 2010. On day 1 of experiment, F1(DBA×BALB/c) mice were irradiated with a therapeutic X-ray machine at a dose of 5 Gy. This radiation dose reduced immunoreactivity, but did not cause death of mice for 6 months and allowed keeping the animals and conducting tests under conventional vivarium conditions. During transplantation, control tumor cells without human PD-L1 were injected subcutaneously into the left fank, while cells expressing PD-L1 were symmetrically injected into the right fank. Tumor volume *(V)* was calculated by formula: *V=(LWH)0.52*, where *L* is the tumor length, *W* is the tumor width, and *H* is the tumor height [16]. Biomarker retention on tumor cells was checked 13-18 days after injection. The mice were sacrificed, tumor nodes were removed, and cells were isolated using BDTM Medimachine System device and Medicon replaceable modules, 50 μm (BD Biosciences, USA). Cell suspensions were filtered using 70 μm Filcon filter (BD Biosciences, USA), cells were washed by centrifugation in phosphate-buffered saline containing 3% fetal bovine serum and 0.1% sodium azide. Human PD-L1 expression was determined immediately after removal and after culturing for 2 weeks without puromycin (selective antibiotic drug). Percentage of PD-L1^+^ cells and fuorescence intensity (which reflects the density of biomarker molecules on membranes) were measured by flow cytometry.

### Biodistribution

Specificity of radioconjugate binding *in vivo* was assessed by direct radiometry after intravenous injection of isotope-labeled nanoantibodies (0.6 MBq/mice). Mice were sacrificed 1, 4, 24, and 48 h after the injection of radiopharmaceuticals, tumors were removed, and their radioactivity was identified using Triathler radiometer (Hidex Oy, Finland). Radionuclide accumulation per 1 g of tissue was calculated as a percentage of the injected activity.

### Statistical analysis

Statistica 10.0 software package was used. The data are represented as a median [Q1; Q3] due to the small sample size (n<10). The Mann– Whitney U test was used to assess differences between two independent samples (p<0.05). All results were performed in at least triplicate.

## Results

**Figure 1.**
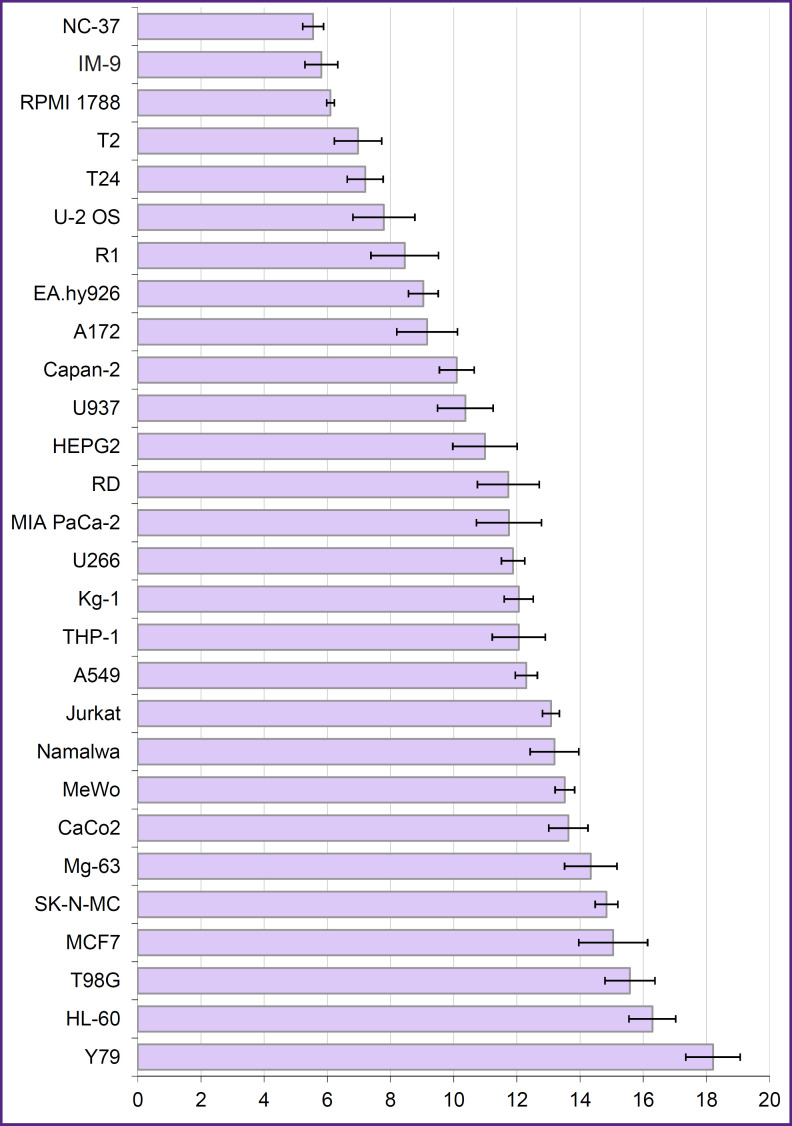
Level of *PD-L1* gene expression in human tumor cell cultures On the abscissa — level of human *PD-L1* gene expression compared to *GAPDH* housekeeping gene (ΔCt); on the ordinate — human tumor cell lines; horizontal segments — standard deviations. At ΔCt<6.5, gene activity was rated as high, ΔCt>13.0 value corresponded to a low or absent gene activity

The expression of *PD-L1* gene was assessed by real-time polymerase chain reaction in 28 human tumor cell lines including epithelial, neuronal, and connective tissue-derived tumors (Figure 1). Over a half of the studied tumors expressed *PD-L1* gene; the highest transcription level was identified in B-lymphoblastoid cell lines NC-37, IM-9, and RPMI 1788. NC-37 cells were used as a source of RNA to obtain cDNA encoding the PD-L1 biomarker.

**Figure 2.**
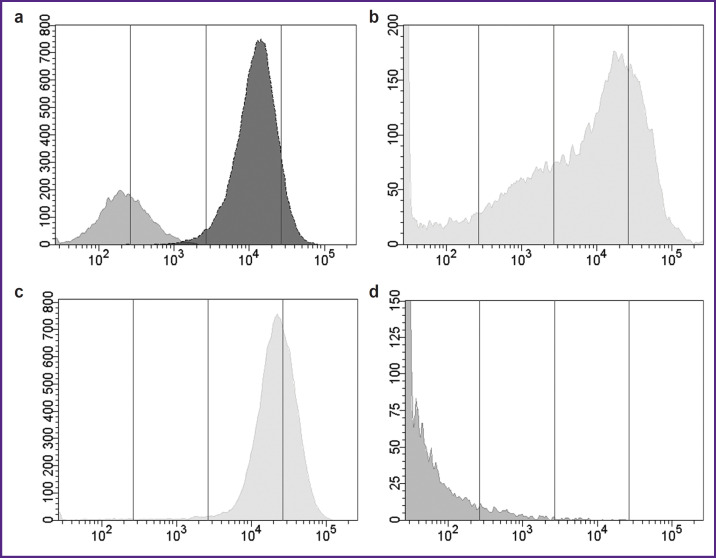
Flow cytometry identification of the cell fraction with membrane human PD-L1: (a) in cultures of NC-37 lymphoblastoid cells (light gray graph) and CT26-PD-L1 cells (dark gray graph); (b) in a suspension of tumor cells extracted on the 13^th^ day after subcutaneous injection of 5 million CT26-PD-L1 cells into F1(DBA×BALB/c) mice; (c) on tumor cells obtained from F1(DBA×BALB/c) mouse after two-week cultivation; (d) in a suspension of regressing tumor cells extracted on the 13^th^ day after subcutaneous injection of 5 million CT26-PD-L1 cells to BALB/c mice. On the abscissa — fluorescence intensity (relative units); on the ordinate — cell number

Primers aimed to amplify cDNA fragment encoding PD-L1 protein full-length membrane form were designed based on the annotated nucleotide sequence NM_014143.4 (GenBank NCBI, USA). The recipient cells of human *PD-L1* gene were CT26 tumor cells from the colon carcinoma of BALB/c mice. The amplified sequence of human PD-L1 cDNA was introduced into CT26 cells by means of retroviral transduction. The transduced cells were selected by adding puromycin to the growth medium. The cells of the resulting strain, CT26-PD-L1, grew as a monolayer and had fibroblast-like morphology; the culture doubling time was 33.50±5.07 h. These parameters were similar to parental CT26 cells. *PD-L1* gene activity in CT26-PD-L1 cells (Δ*Ct*=0.36) corresponded to the activity of the housekeeping gene (*GAPDH* of mice) and was tenfold higher than in NC-37 lymphoblastoid cells used as the gene donors (Δ*Ct*=5.5). According to the flow cytometry data, CT26-PD-L1 strain contained 99.8% cells expressing human PD-L1; in NC-37 culture this biomarker was detected in 40.4% of cells. The median cell fluorescence intensity, an indicator of antigen density on cell membrane, was by two orders of magnitude higher in genetically modifed CT26-PD-L1 cells compared with NC-37 cells (Figure 2 (a)). Parental murine cells (CT26) did not carry membrane PD-L1 molecules and served as a negative control.

To assess the stability of human biomarker expression on CT26-PD-L1 cells, the 4^th^ passage cell population was divided into two parts. The frst part was cultured and seeded in a medium containing puromycin (selective agent), whereas the second part was cultured without the antibiotic drug. At the 16^th^ passage *PD-L1* gene activity and membrane expression in both cell cultures corresponded to the initial parameters thus indicating the stability of the strain.

To create a tumor model expressing human PD-L1, CT26-PD-L1 cells were injected subcutaneously to intact BALB/c mice or sublethal irradiated F1(DBA×BALB/c) mice. 9–13 days after injection of 5 million cells, palpable tumor masses were formed in 100% of irradiated F1(DBA×BALB/c) mice. In BALB/c mice the efficacy of CT26-PD-L1 cell inoculation was significantly lower; at this time point, progressive tumor growth was detected only in 23% of mice. In other 77% of mice by the 13^th^ day after cell injection previously formed tumors regressed or there were no tumors (69 and 8%, respectively). In the case of tumor progression in BALB/c mice, the volume of neoplasms on the 13^th^ day was 133.6 [74.9; 243.4] mm^3^ that was significantly less than the tumor volume in irradiated F1(DBA×BALB/c) mice — 898.6 [393.1; 1,755.0] mm^3^.

Cell suspensions from tumors of F1(DBA×BALB/c) mice contained 43.7–49.8% cells expressing membrane human PD-L1 (Figure 2 (b)). Cell suspensions from murine tumors were explanted into *in vitro* cultures, and the number of cells with membrane human PD-L1 was re-assessed two weeks later. The content of PD-L1^+^ cells increased to 98.5% accompanied by recover of fluorscence intensity intrinsic for the original cultured cells (Figure 2 (c)). These results provide evidence of stable PD-L1 expression by genetically modifed cells in our *in vivo* model. In regressing tumors of BALB/c mice only 4.2% of cells had the membrane biomarker (Figure 2 (d)). Thus, the stable retention of tumor growth with human PD-L1 expression demonstrated the advantage of sublethally irradiated F1(DBA×BALB/c) mice compared with intact BALB/c mice.

**Figure 3.**
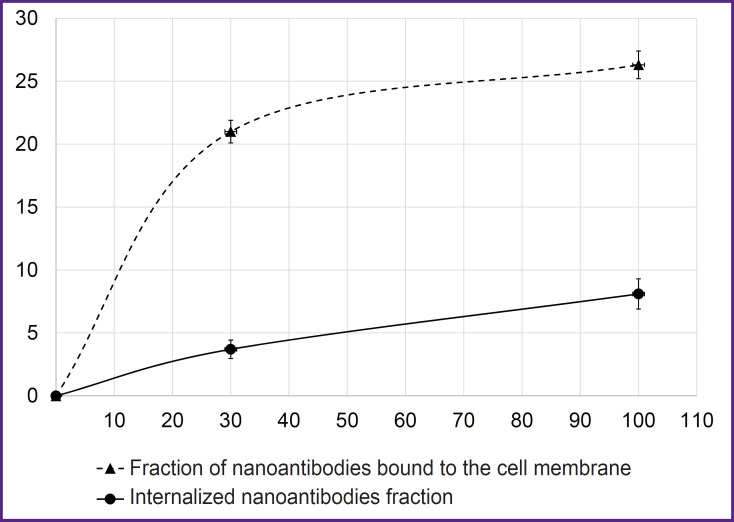
Internalization and binding of [^68^Ga]Ga-VHH-PD-L1 by CT26-PD-L1 cells On the abscissa — cell incubation time with radioconjugate (min); on the ordinate — cell-binded radioactivity (%); vertical segments — standard deviations

The possibility of using genetically modified cells to assess the specificity of binding and internalization of affinity molecules *in vitro* was studied using the [^68^Ga]Ga-VHH-PD-L1 radioconjugate. Cells were incubated with radionuclide-labeled nanoantibodies for 30 or 100 min at 37°C. The value of cell-binded radioactivity increased by 2.2 times from 30 to 100 min of incubation, indicating its internalization (Figure 3). Specificity of binding of [^68^Ga]Ga-VHH-PD-L1 to PD-L1 expressed on CT26-PD-L1 cell membranes was confirmed by the background values detected for control CT26 cells (≤0.5%).

**Figure 4.**
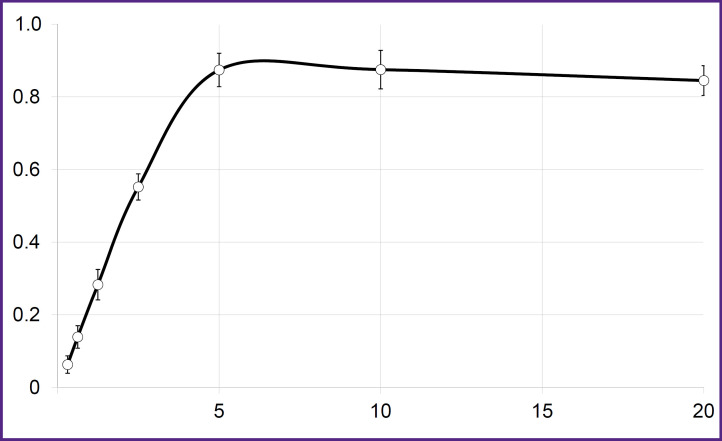
Assessment of the target-binding fraction of [^68^Ga]Ga-VHH-PD-L1 with CT26-PD-L1 cells On the abscissa — cell concentration (million/ml); on the ordinate — cell-binded radioactivity/total radioactivity; vertical segments — standard deviations

CT26-PD-L1 strain was tested *in vitro* in order to estimate the fractions of radiolabeled nanoantibodies that retained PD-L1-binding ability. For this purposes ^68^Ga or ^177^Lu-labeled radioconjugates were used. In this case, cells were incubated with radioconjugates at 4°C to avoid internalization. The results of testing ^68^Ga-labeled anti-PD-L1 nanoantibodies are shown as an example (Figure 4). The binding of all radiolabeled molecules that retained specificity was reached at CT26-PD-L1 cells concentration of 5 million/ml. It corresponded to a plateau in the graph showing the dependence of the ratio of cell-bound radioactivity to the total radioactivity introduced into the sample. Thus, the immunoreactive fraction of radioconjugate was 87.4%, indicating significant preservation of the antigen-binding properties of anti-PD-L1 nanoantibodies during the conjugation.

**Figure 5.**
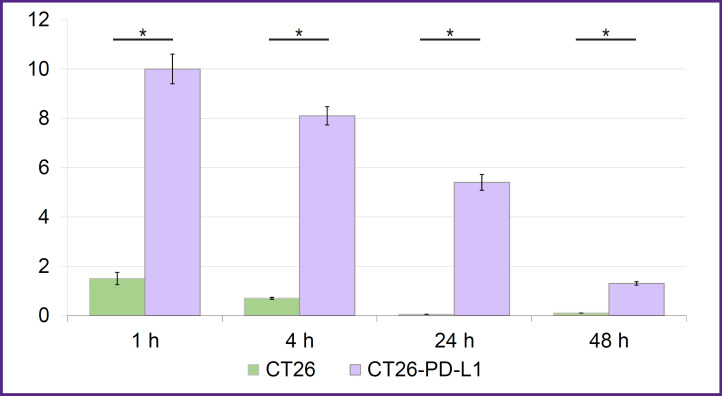
Assessment of the [^177^Lu]Lu-VHH-PD-L1 specificity *in vivo* On the abscissa — time from radioconjugate injection to tumor removal; on the ordinate — radioconjugate accumulation (%) of the injected dose/g tissue; vertical segments — standard deviations; * p<0.001. The test was performed on sublethally irradiated F1(DBA×BALB/c) mice on the 12^th^ day after transplantation of CT26-PD-L1 or CT26 cells

To assess the *in vivo* binding specificity of the conjugates, affine to human PD-L1, CT26-PD-L1 cells were injected into sublethally irradiated F1(DBA×BALB/c) mice. The results of testing [^177^Lu]Lu-VHH-PD-L1 in this model (Figure 5) demonstrated its high specificity. It rapidly accumulated in the tumor bearing human PD-L1 and remained there for 2 days. In the control CT26 tumor the radioconjugate’s accumulation corresponded to the background value.

## Discussion

PD-L1 membrane protein expressed by antigen-presenting cells is a so-called immune checkpoint molecule since its binding to the PD-1 receptor on T-lymphocytes normally prevents the development of uncontrolled immunological reactions. Scientific data [6, 33, 34] as well as our results (see Figure 1) indicate that epithelial, neuronal, and connective tissue-derived tumor cells have an active *PD-L1* gene. Increased PD-L1 expression on the membrane of tumor cells of various origin and its dominant role in suppressing antitumor immune response make it an overall target for treatment of a wide range of malignant neoplasms [5–7].

Currently, three anti-PD-L1 drugs are approved for use in clinical practice: atezolizumab, durvalumab, and avelumab. All of them are monoclonal antibodies that prevent PD-L1 binding to PD-1. Pharmacological action of the drugs is directed to antitumor immunity reactivation. Avelumab also induces tumor cell lysis by activating antibody-dependent cell cytotoxicity. However, a significant number of patients do not respond to this immunotherapy. The reasons for ineffectiveness include lack of cytotoxic T-lymphocytes and NK-cells in tumors, primary or secondary resistance to PD-L1 inhibitors due to other immunosuppressive mechanisms [12, 35]. These facts stimulate development of new drugs aimed primarily at the direct destruction of tumor PD-L1-expressing cells. At the same time researchers investigate approaches to determine the level of PD-L1 expression in malignant neoplasms to select patients subjected for therapy with PD-1/PD-L1 immune checkpoint inhibitors or PD-L1-targeted cytotoxic drugs. Traditionally used immunohistochemistry is limited to assessment of single biopsy tumor fragments. Meanwhile high heterogeneity in PD-L1 expression is seen even within a single tumor node [6, 7, 36, 37]. Molecular imaging methods allow assessment of the biomarker distribution in patient’s body.

In recent years targeted nuclear medicine has become increasingly popular. It uses radioconjugates that specifically bind molecular targets on tumor cells for cancer diagnosis and treatment. Several anti-PD-L1 radiopharmaceuticals intended for diagnostics and therapy are being developed or are at the clinical trial stage [18–22, 38–47].

Immune checkpoint inhibitors require mandatory preclinical assessment of immune response reactivation. On the contrary, development of radioconjugates requires confirmation of the stability of target specificity and, in case of a therapeutic drug, testing its cytotoxic effect on tumor cells with biomarker.

Along with human tumor cell lines, genetically engineered cells created on the basis of murine tumor cells are used abroad as cell models for testing radioconjugates that target PD-L1 biomarker [18–22]. We continued to study the potential of humanized murine tumor cells as model objects for *in vivo* and *in vitro* testing of anti-PD-L1 pharmaceuticals. The genetic engineering approach resulted in creating the CT26-PD-L1 cell strain with over 99% cells expressing membrane human PD-L1 with high density. These cells stably expressed PD-L1 during long-term cultivation *in vitro*. Assessment of cells tumorigenicity in immunocompetent syngeneic BALB/c mice revealed progressive tumor growth in 23% of cases. Other animals demonstrated tumor regression or no neoplasms during the entire observation period. Low tumorigenicity of cells in intact mice could relate to moderate amino acid sequence homology of human and murine PD-L1 — approximately 70% [1]. Development of an immune response to human PD-L1 in intact animals was confirmed by preferential selection of cells with reduced or lost human antigen demonstrated by a significantly lower number of PD-L1^+^ cells in regressing tumors compared to progressively growing tumors (4.2 and 50.0% PD-L1^+^ cells, respectively). In our study sublethal X-ray irradiation of F1(DBA×BALB/c) mice was used for partial immunosuppression; the absorbed dose was 5 Gy. This allowed to reduce murine immunoreactivity leading to human PD-L1 expressing tumor growth in 100% of animals. Tumor growth in such mice was not accompanied by an increase in the number of cells with lost or reduced expression of the human biomarker. It was supported by the data obtained after *in vitro* cultivation of cells from growing tumors. Simultaneously the level of immunosuppression induced by sublethal irradiation allowed maintaining these animals and conducting experiments in a conventional vivarium. This is the advantage of our model compared with ones using nude, SCID, and other mice. We had no opportunity to inoculate our CT26-PD-L1 cells into immunodeficient BALB/c nude mice. However, publications showed that humanized murine cells expressing human PD-L1 had been implanted into immunodeficient [20–22], humanized [18, 19, 24] or SPF mice [17, 23, 25]. It should be noted that SPF mice, like other previously mentioned animals, require maintenance in a barrier-type vivarium and are not fully immunocompetent [48, 49]. This study has supplemented the list of murine models that do not require special maintenance conditions.

Assessment of the resulted CT26-PD-L1 strain for testing PD-L1-affinity agents *in vivo* and *in vitro* was conducted using targeted radioconjugates based on nanoantibodies created in the A.M. Granov Russian Research Center for Radiology and Surgical Technologies.

Efficacy of using CT26-PD-L1 cells as cell models to assess the specificity of radioconjugates *in vitro* was tested using nanoantibodies labeled with ^68^Ga. The specificity of the interaction of the radioconjugate with CT26-PD-L1 cells was confirmed by an increase in radioactivity together with the growth in the number of cells in the sample and absence of radioconjugate binding to control CT26 cells. High biomarker density on the cells allowed to achieve saturation of the radioconjugate molecules with target molecules and to quantify the content of the fraction of molecules capable of binding PD-L1 after the radionuclide attachment.

Natural recirculation of PD-L1 molecules between the cell membrane and inner cell compartments allows to use PD-L1-affinity agents, whose action is associated with intracellular localization for diagnostic and therapeutic purposes. In this case, evaluation of pharmaceuticals’ internalization by tumor cells becomes a key stage of their development. Agents that penetrate the cell are retained in the tumor node longer leading to increased tumor contrast against the background during diagnostic procedures, for instance, positron emission tomography or single-photon emission computer tomography [13, 45, 47, 50]. The information on the use of humanized murine cells to assess internalization of PD-L1-targeted radioconjugates was not found. With the help of [^68^Ga]Ga-VHH-PD-L1 we demonstrated that CT26-PD-L1 cells can internalize the biomarker.

When radioimmunoconjugates were injected into mice, radionuclides accumulated in tumors from transplanted CT26-PD-L1 cells but not from control CT26 cells. This result confirmed suitability of the proposed tumor model for testing *in vivo* specificity of pharmaceuticals that target human PD-L1.

## Conclusion

CT26-PD-L1 cells created by means of CT26 murine carcinoma retroviral transduction had high-density of human PD-L1 molecules on the membrane. Our cells were considerably stable during cultivation and formed tumors after transplantation into intact BALB/c or sublethally irradiated F1(DBA×BALB/c) mice. Cell trials as a biological model for *in vitro* and *in vivo* assessment of PD-L1 targeted agents were conducted using radioimmunoconjugates that contained recombinant single-domain antibodies to PD-L1 and ^68^Ga or ^177^Lu radionuclides. *In vitro* experiments demonstrated the possibility of using CT26-PD-L1 cells for development of anti-PD-L1 pharmaceuticals, whose action is associated with internalization. Due to high density of target molecules on the membrane, CT26-PD-L1 cells allowed to confirm the specificity of the pharmaceuticals and quantify the target-binding fraction of conjugates in a single test. When radioimmunoconjugates were injected into mice, radionuclides accumulated in CT26-PD-L1 tumors but not in control CT26 tumor cells. A significant advantage of the created tumor model was the possibility to conduct *in vivo* testing of anti-PD-L1 pharmaceuticals specificity on animals under conventional vivarium conditions.

As far as we know, the cells obtained in this study are the first genetically engineered cells designed to assess anti-PD-L1 therapeutic and diagnostic agents in Russia. This cell model can be used to measure specific activity of not only radioimmunoconjugates but also other pharmaceuticals with affinity to human PD-L1. In general, our results prove the efficacy of humanized cells for the targeted agents’ testing.
